# A review of pursuit and saccadic eye movements and their utility in stroke

**DOI:** 10.3389/fstro.2023.1247326

**Published:** 2023-09-18

**Authors:** Elizabeth Fracica, David E. Hale, Daniel R. Gold

**Affiliations:** ^1^Department of Neurology, The Johns Hopkins Hospital, Baltimore, MD, United States; ^2^Division of Neuro-Visual and Vestibular Disorders, Department of Neurology, The Johns Hopkins Hospital, Baltimore, MD, United States

**Keywords:** AVS, saccade, smooth pursuit, stroke, HINTS

## Abstract

The head impulse-nystagmus-test of skew (+ hearing) or HINTS+ exam is a well-established clinical bedside test used in evaluating whether patients with the acute vestibular syndrome have features concerning for a central etiology (e.g., stroke). There are other components of the ocular motor exam that are helpful in the acute setting, including smooth pursuit and saccades. We discuss the anatomy and physiology of the saccade and smooth pursuit pathways from the cortex to the infratentorial region in the context of anterior and posterior circulation strokes in general but with a particular emphasis on distinct vestibular stroke syndromes. For each stroke localization, we review the vascular supply and the expected findings on the HINTS+ exam and correlate this with the expected findings on the smooth pursuit and saccade exams to aid in bedside diagnosis.

## 1. Introduction

Stroke remains a leading cause of disability and death both in the United States and globally (Tsao et al., 2023). An estimated 20–25% of strokes occur in the posterior circulation, of which the majority are ischemic strokes (Merwick and Werring, 2014). Dizziness is a common presentation of posterior circulation stroke (PCS), occurring in up to 50% of cases (Searls, 2012; Kim et al., 2022). Unfortunately, the diagnosis of dizziness is challenging and is more often due to benign causes (Newman-Toker et al., 2008) such that there is a risk of overutilizing resources (especially computed tomography and magnetic resonance imaging [MRI]), as well as a risk of misdiagnosis (Newman-Toker, 2016). Dizziness as a presenting symptom of stroke increases the misdiagnosis risk 14-fold compared to motor symptoms, and vestibular strokes are missed in 35% of cases (Kerber et al., 2006; Tarnutzer et al., 2017). Furthermore, stroke was identified as the number one cause of misdiagnosis in a systematic review of the literature on missed diagnoses in the emergency department (Newman-Toker et al., 2022). A minority of patients with PCS present with isolated dizziness without any additional exam findings, although up to 50% of vertebrobasilar transient ischemic attacks (TIAs) may present with isolated dizziness (Kerber et al., 2006; Paul et al., 2013).

The head impulse-nystagmus-test of skew (+ hearing) or HINTS+ exam is a critical tool in evaluating the patient with acute vestibular syndrome (AVS), defined as acute onset vertigo with spontaneous nystagmus, nausea, vomiting, gait unsteadiness, and intolerance of head motion (Bisdorff et al., 2015). The HINTS exam is highly specific and sensitive for the detection of central causes of the AVS in the hands of subspecialists and performs better than MRI in the first 24 h (Kattah et al., 2009). However, there have been barriers to its broader adoption, including difficulty with the technique and the interpretation of the HINTS+ exam, leading to reduced sensitivity and specificity among non-specialists (Ohle et al., 2020). The use of the HINTS+ exam has also been limited during tele-stroke consultations. Furthermore, the use of HINTS+ exam in cases where patients lack spontaneous nystagmus is less clear. Kim et al. studied patients with PCS with acute dizziness who had initially negative MRI and found HINTS could only be applied to 40% of patients due to a lack of spontaneous nystagmus at the time the patient was assessed (Kim et al., 2022).

In this setting, there have been additional efforts to study other symptoms and signs that may aid in PCS diagnosis. PCS may also present with acute dizziness accompanied by severe imbalance with truncal ataxia, whereas those with peripheral causes were less severely affected. Carmona et al. found the presence of severe truncal ataxia characterized by falling at upright posture (grade 3) in combination with HINTS was 100% specific for a central lesion, while grade 1 or 2 ataxia was seen in both peripheral and central lesions (Carmona et al., 2016; Kattah et al., 2022). Thus, severe imbalance and truncal ataxia may be additional valuable predictors of PCS, especially in combination with central HINTS+ exam (Kerber et al., 2006; Carmona et al., 2016). To capture patients who present with acute dizziness and imbalance without spontaneous nystagmus, acute imbalance syndrome has been discussed in the literature (Machner et al., 2021). Beyond the HINTS+ exam and the presence of ataxia, dizzy patients with PCS may present with additional ocular motor and vestibular exam findings, which are highly localizing in some cases (Kim et al., 2017).

The HINTS+ exam is very accurate in the hands of experts, but not everyone is comfortable or as experienced, which can lead to missing strokes presenting with AVS. In our experience, one common barrier seems to be the aversion to performing the necessary quick head movements in the head impulse test due to fear of exacerbating the patient's dizziness (and the wrath that might ensue) or patient pushback or refusal. The nystagmus in central AVS can sometimes be so subtle that it is masked by fixation and when providers are not comfortable with testing for fixation-removed nystagmus, it can be missed. These and other barriers may explain some of the gaps observed in the sensitivity and specificity of the HINTS+ exam when comparing experts with non-specialists and trainees. Because there is often diagnostic uncertainty regarding the interpretation of findings on the HINTS+ exam due to a lack of provider confidence in performing elements of the HINTS+ exam, there is potential value in identifying additional clinical exam findings that may facilitate the accurate diagnosis of stroke in patients presenting with AVS.

There are several characteristic patterns on smooth pursuit and saccade testing that can be seen in stroke syndromes (including those presenting with AVS). While these patterns are quickly recognizable among specialists, it is rare that neuro-otology and neuro-ophthalmology attendings can be present at the bedside in the emergency department. Testing saccades and smooth pursuit function is a simple and non-invasive window into the brain that may overcome some of the barriers that can be encountered with the head impulse test, in particular. In cases where the HINTS+ exam is clearly central, there is no need for additional testing. It is in the cases of diagnostic uncertainty where promptly recognizing the patterns of saccadic and smooth pursuit abnormalities described in this article may be the difference between a missed stroke or the triumphant rescue of an ischemic penumbra.

We aim to help the reader understand how these pathways become clinically relevant at the bedside and how to interpret these exam findings in the context of stroke syndromes affecting various parts of each pathway. Our hope is that this additional knowledge of smooth pursuit and saccade function may, in a few select cases, improve diagnostic accuracy and serve to complement the examiner's existing knowledge of the HINTS+ exam when evaluating dizzy patients with a concern for stroke.

In this article, we provide a comprehensive review of strokes occurring within the anterior and posterior circulation that can lead to abnormalities in saccades and smooth pursuits where dizziness is the major or, at least, an associated symptom. Although the use of saccades and smooth pursuit has not been studied in the acute assessment of suspected ischemic stroke, abnormalities in their assessment can provide localizing value. We review how to perform and interpret the bedside assessment of saccades and smooth pursuits. We also review the anatomy of the saccade and smooth pursuit pathways and discuss stroke syndromes in this context. While the focus is on strokes that affect the central vestibular pathways (and thus present with dizziness), we also review key neighboring structures in the brainstem that may be involved in larger stroke lesions that lead to characteristic or highly localizing smooth pursuit and saccadic abnormalities. Finally, we provide a comprehensive review of the expected pattern of HINTS+ exam findings for lesions throughout the posterior circulation and correlate this with the expected pattern of smooth pursuit and saccade findings, vascular territory, and any other characteristic neurologic exam findings.

## 2. Smooth pursuit

### 2.1. Purpose

The function of smooth pursuit is to keep a slowly moving target stable on the fovea of each eye. If smooth pursuit cannot keep up with a visual target (i.e., low pursuit gain, which is defined as the ratio of eye movement velocity to target velocity), saccades will be used to help catch up to the visual target, hence the choppy or saccadic appearance when pursuit is impaired.

### 2.2. Assessing smooth pursuit

When testing smooth pursuit, the examiner should use a small target for fixation and slowly move the target in the horizontal and vertical directions at approximately 10–15 degrees/s. The target should be just shy of arm's length to allow the examiner to carefully observe the eye movements, which will be smooth when normal (Normal Smooth Pursuit VIDEO; Gold, 2016). When abnormal, pursuit is interspersed with saccades and appears “choppy,” with varying degrees of severity. There should be mention of whether the smooth pursuit function is symmetric and whether there are differences in vertical pursuit in comparison with horizontal pursuit (VIDEO Abnormal Pursuits; Gold, 2018).

Accurate assessment of smooth pursuit will be limited in the patient who is encephalopathic, delirious, lethargic, or otherwise not engaged. In these instances, the examiner can try to assess the smooth pursuit component or “slow phase” of the nystagmus that can be generated with an optokinetic stimulus (such as an optokinetic drum), which may provide enough of an additional stimulus to improve participation in some patients.

### 2.3. Anatomy of the horizontal smooth pursuit pathway

**Figure 1:**
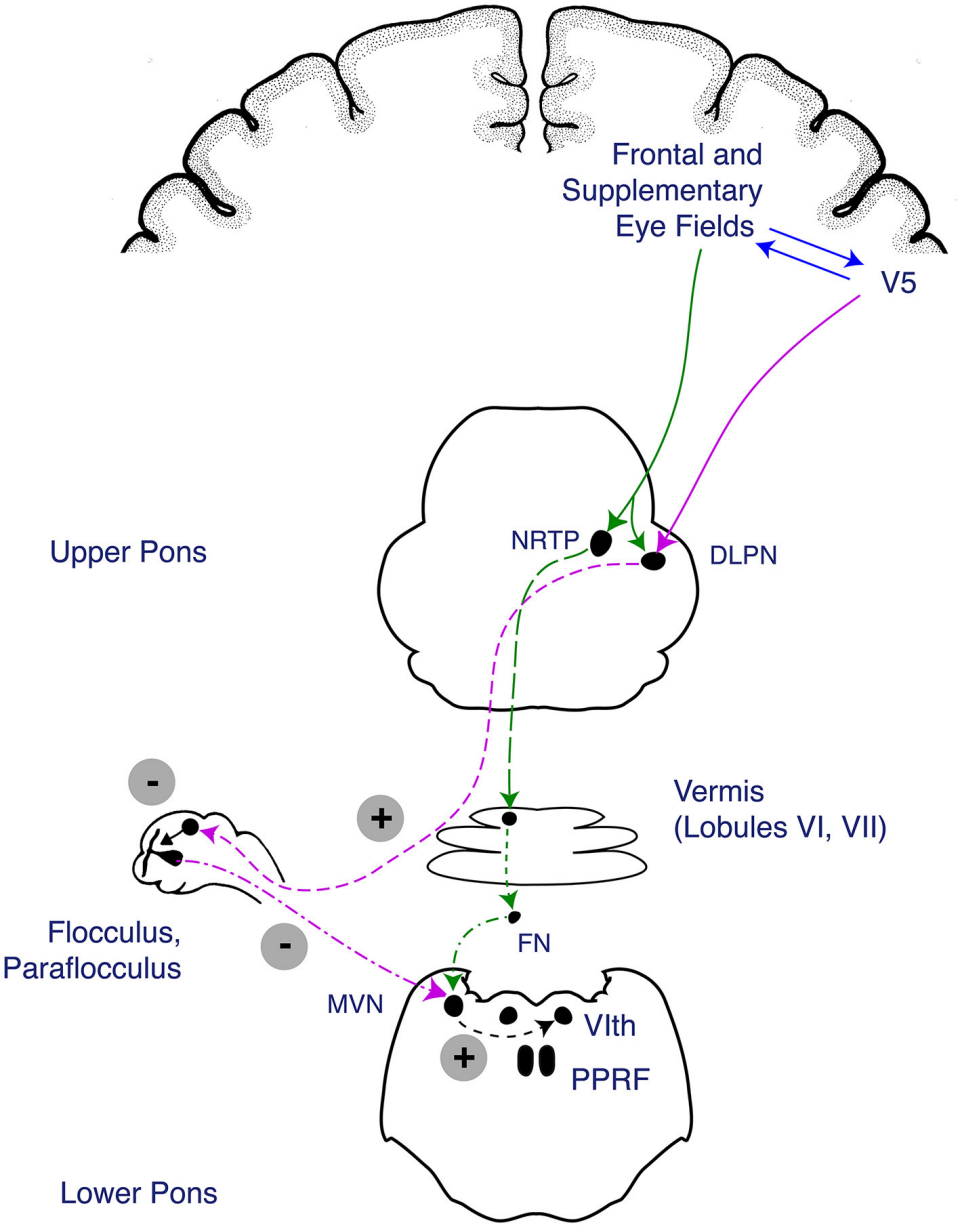
Anatomy of the smooth pursuit pathway. The smooth pursuit pathway enables tracking of a slow-moving target while keeping the target fixed on the fovea. The smooth pursuit pathway receives afferent input from the retina where retinal image motion is detected and carried through the afferent optic pathway from the optic nerve to the lateral geniculate nucleus of the thalamus and up to the medial and superior temporal cortices (MT/MST), which process image motion before projecting to the frontal eye field (FEF) (Heide et al., 1996; Leigh and Zee, 2015). Descending fibers involved in the *initiation* of smooth pursuit travel from the FEF to the medial pons (nucleus reticularis tegmenti pontis [NRTP]) before projecting through the bilateral middle cerebellar peduncles (MCPs) to the cerebellar vermis and fastigial nucleus (FN). Descending fibers involved in *maintenance* of smooth pursuit travel from the cortical MT/MST to the dorsolateral pontine nucleus (DLPN), which travel to the cerebellar flocculus and paraflocculus of the cerebellum, then to the medial vestibular nucleus (MVN). The fibers from the DLPN and NRTP project to cerebellar structures (vermis, flocculus, and paraflocculus), which eventually act on the MVN to trigger a horizontal gaze deviation through activation of the abducens nucleus is the final endpoint of the pathway. Figure reproduced with permission from Liu, Volpe, and Galetta's Neuro-Ophthalmology: Diagnosis and Management. 3rd Edition. Copyright 2018, Elsevier.

The smooth pursuit pathway involves a network of cortical connections that are dependent on afferent input of retinal image motion, which travels through the geniculo-striate system to the medial and superior temporal cortices (MT/MST), which are important for processing image motion before projection to the frontal eye field (FEF) (Heide et al., 1996; Leigh and Zee, 2015). From the FEF, the pathways for horizontal smooth pursuit *initiation* project to pontine nuclei in the medial pons (nucleus reticularis tegmenti pontis), which then send fibers out bilaterally through the middle cerebellar peduncles (MCPs) to the cerebellum (dorsal vermis [DV] and fastigial nucleus [FN]). Once smooth pursuit is initiated, the brain is continually tracking foveal slip, which can occur with changes in velocity or acceleration of the target, leading to new estimates of smooth pursuit velocity. The pathway controlling smooth pursuit *maintenance* (involving the tracking of a fixed-velocity target and adjustments occurring when there is slippage of the target off the fovea) travels from the MT/MST to the dorsolateral pontine nucleus to the paraflocculus of the cerebellum and then to the vestibular nucleus and, finally, to the ocular motor neurons. There are additional inputs from the inferior olive, the medullary nucleus prepositus hypoglossi (NPH), and the medial vestibular nucleus (MVN), as part of the accessory optic pathway (Leigh and Zee, 2015). Thus, it becomes apparent that lesions causing impairment of smooth pursuit might localize supratentorially to the FEF and the MT/MST and infratentorially to the medial or lateral pons, the medial medulla (affecting the MVN), the middle and inferior cerebellar peduncles (MCP and ICP), or the paraflocculus (tonsil) or vermis of the cerebellum. Vertical smooth pursuit pathways in the brainstem and cerebellum are less clearly understood, and isolated vertical pursuit impairment is unlikely to occur in the setting of a stroke, so it is not covered here (Leigh and Zee, 2015). For a high-level summary of where deficits in the smooth pursuit may localize, (see Table 1). The smooth pursuit pathway is illustrated in Figure 1.

**Table 1:** High-level localization of smooth pursuit deficits.

**Supratentorial**	**Infratentorial**
Frontal eye fields	Pons (medial or lateral)
Medial and superior temporal cortices	Medial medulla (medial vestibular nucleus)
Subcortical structures including basal ganglia, internal capsule, and thalamus	Middle and inferior cerebellar peduncles
	Cerebellar vermis, flocculus, and paraflocculus (tonsil)

### 2.4. Anterior circulation strokes associated with abnormal smooth pursuit

**Vasculature**: anterior circulation (middle cerebral artery [MCA], anterior cerebral artery [ACA], and internal carotid artery [ICA]).

**Key Structures:** FEF, parietal and occipital cortex, internal capsule, basal ganglia, and thalamus.

The smooth pursuit pathway is broadly distributed such that lesions in many different supratentorial areas have been associated with horizontal smooth pursuit deficits (Lekwuwa and Barnes, 1996). Vertical smooth pursuit tends to be intact with unilateral supratentorial lesions. Patients with cortical strokes affecting the FEF, the posterior parietal cortex, the occipital cortex, and the surrounding white matter may result in impaired smooth pursuit. Smooth pursuit is also impaired in patients with strokes affecting subcortical regions, including the basal ganglia, the internal capsule, and the thalamus (Lekwuwa and Barnes, 1996).

Smooth pursuit is classically impaired ipsilesionally to a supratentorial stroke with contralesional saccade abnormalities (Kudo et al., 2021). Unilateral lesions of the posterior limb of the internal capsule, the basal ganglia, the FEF, and the posterior parietal cortex have all been associated with ipsilesional deficits in smooth pursuit gain (Morrow and Sharpe, 1990; Rivaud et al., 1994; Caplan and van Gijn, 2012). In one review, patients with the most pronounced ipsilesional deficits in smooth pursuit primarily had subcortical lesions involving the internal capsule and basal ganglia (Lekwuwa and Barnes, 1996).

Supratentorial lesions can also impair smooth pursuit function bilaterally, which can be symmetric or asymmetric (i.e., more severe ipsilesionally) (Park et al., 2023). Bilateral pursuit impairment was observed in lesions of the posterior parietal cortex, the FEF and its surrounding white matter, and the thalamus (Lekwuwa and Barnes, 1996). A subsequent study evaluating 23 patients with unilateral cerebral lesions found that the regions most responsible for asymmetric bidirectional deficits localized to Brodmann area 19, part of the occipital lobe cortex, and Brodmann area 39 in the parietal cortex, with their areas of surrounding white matter (Sharpe and Morrow, 1991).

Because the smooth pursuit system is broadly distributed throughout the brain and receives cortical inputs from the FEF and the parietal, temporal, and occipital lobes, supratentorial lesions in many regions within this network can produce smooth pursuit deficits, although typically this is only in the horizontal plane. Vertical smooth pursuits tend to be intact in unilateral supratentorial causes of smooth pursuit impairment. While smooth pursuit may be abnormal in supratentorial lesions, deficits in smooth pursuit function are typically minor in comparison to the more substantial cognitive, language, motor, sensory, and other deficits commonly observed in anterior circulation infarcts.

### 2.5. Posterior circulation strokes associated with abnormal smooth pursuit

**Vasculature:** anterior inferior cerebellar artery (AICA), posterior inferior cerebellar artery (PICA), and basilar, vertebral, and anterior spinal artery (ASA).

An AICA territory stroke (supplying the dorsolateral pons, the MCP, and the flocculus) may produce a smooth pursuit abnormality, as these structures are all involved in the smooth pursuit pathway. The paraflocculus (tonsil) and the vermis are supplied by the PICA and are both involved in smooth pursuit. Strokes affecting the medial pons (basilar) and the medial medulla (vertebral and ASA) can also cause impaired smooth pursuit.

#### 2.5.1. Middle cerebellar peduncle

The MCP (located in the caudal lateral pons) and the dorsolateral pontine nuclei (deep cerebellar nuclei) are important structures involved in smooth pursuit (Kato et al., 1991; Kim and Kim, 2019; Shemesh and Zee, 2019). Smooth pursuit deficits arising from MCP lesions are typically severe and bilateral because each MCP receives fibers from ipsilateral and contralateral pons (Kim and Kim, 2019). In one study of 23 patients with isolated MCP stroke, patients consistently demonstrated bilaterally impaired smooth pursuit (Kim and Kim, 2019). There is one report of inappropriate torsion during vertical smooth pursuit in a patient with an MCP lesion (FitzGibbon et al., 1996). Additional neurologic signs of MCP stroke include dysarthria, ataxia, hemiparesis, and hemisensory loss. If there is extension into the dorsolateral pons, there may be trigeminal facial sensory loss, abducens paresis, and facial weakness ipsilesionally due to involvement of the fifth, sixth, and seventh cranial nerve nuclei or tracts, respectively (Caplan and van Gijn, 2012). Strokes affecting the dorsolateral pons (sparing the MCP) typically lead to ipsilateral pursuit deficits (Thier et al., 1991; Gaymard et al., 1993). The pattern of additional deficits expected on the HINTS+ exam and the neurological exam are described in Table 2 for MCP and dorsolateral pontine lesions.

**Table 2:** HINTS “plus” patterns in the acute vestibular syndrome based on location.

**Anatomic structure**	**Vascular supply**	**Head impulse test**	**Nystagmus**	**Test of skew (and other OTR features)**	**“Plus” (hearing loss)**	**Smooth pursuit (SP)**	**Saccades (SAC)**	**Other neurologic or ocular motor signs**	**Other general neurologic signs**
**Labyrinth/8**^**th**^ **cranial nerve**	AICA	++	Spontaneous: ++ contra; Spontaneous upbeat-torsional (from a rostral superior vestibular nucleus lesion in the dorsolateral pons) (Chang et al., 2021); or spontaneous horizontal-torsional nystagmus (isolated 8th fascicular infarct) (Kim and Lee, 2010).	-	Labyrinth: ++ Normal otoscopy and hearing loss present à concern for labyrinthine ischemia	Normal	Normal	Normal otoscopy and hearing loss present a concern for labyrinthine ischemia	Imbalance, gait impairment with truncal ataxia
			GEN: -		Nerve: ^−^ (vestibular neuritis)				
**Medial medulla**	Vertebral A, ASA	+ (MVN–ipsi; NPH–contra) MVN-NPH lesions à HC VOR loss	Spontaneous: + (upbeat)	+/–	_	May see bilaterally abnormal horizontal smooth pursuit (if MVN-NPH affected)	Contrapulsion, contra hypermetria, ipsi hypometria	MVN-NPH lesions à HC VOR loss and horizontal GEN (e.g., Wernicke's)	Dejerine syndrome—ipsilateral tongue weakness, contralateral hemiparesis, and hemisensory loss (spares face)
			GEN: + (Wernicke's) GEN: +						
**Lateral medulla/inferior cerebellar peduncle**	PICA, vertebral, basilar branches	+ (afferents to MVN)	Spontaneous: +	++ (contra hyper)	-	Contralesional smooth pursuit impairment	Ipsipulsion, ipsi hypermetria, contra hypometria	Ipsiversive ocular tilt reaction	Wallenberg syndrome—dysarthria, ipsilateral face/contralateral hemibody impaired pain/temperature, sensation, ipsilateral ataxia, hiccups, Horner's syndrome
			GEN: +						
**Nodulus and uvula**	PICA	-	Spontaneous: + ipsi	+ (ipsi hyper)	-	Impairment of downward SP	Normal	PAN and/or ^*^central HSN	Severe ataxia and imbalance
			GEN: -						
			Positional: + (apogeo) Other: PAN and/or ^*^central HSN						
**Paraflocculus (Tonsil)**	PICA	-	Spontaneous: + ipsi	+ (ipsi hyper)	-	Asymmetric impairment, worse ipsilesionally	Normal	Cervicomedullary lesions (e.g., Chiari)	Ataxia, Wallenberg syndrome (if due to PICA and lateral medulla is also affected)
			GEN: ++						
			Positional: + (geo)						
**Flocculus**	AICA	+ (contra)	Spontaneous: + ipsi	++ (ipsi hyper)	+ (AICA)	Symmetric impairment, worse ipsilesionally	Normal	DBN with symmetric lesions (e.g., SCA); ^*^central HSN	If additional AICA territory structures affected, may also have ipsilesional hearing loss, ataxia
			GEN: ++						
**Dorsolateral pons/middle cerebellar peduncle (MCP)**	AICA, SCA (dorsolateral pons)	+ (only when 8^th^ N fascicle is involved)	Spontaneous: +	+ (contra>ipsi hyper) Ipsiversive OTR and SVV tilt common	+ [AICA to internal auditory artery supplying labyrinth or due to cochlear nucleus ischemia (AICA)] (AICA)	Severely impaired bilateral horizontal smooth pursuit (MCP lesions)	Normal	Impaired smooth pursuit with MCP lesions; can have ipsi 6th and 7^−^ nerve palsies	Can have ipsilateral facial sensory loss, abducens palsy and facial weakness due to involvement of 5^th^, 6^th^ and 7^th^ CN nuclei or tracts in lateral pons (AICA). Also dysarthria, ataxia, hemiparesis, hemisensory loss (Deplanque et al., 1998; Caplan and van Gijn, 2012).
			GEN: ++						
**Medial pons**	Paramedian basilar A	-	Spontaneous: + (vertical-torsional)	++ (ipsi hyper)	-	MLF lesions can cause mild impairment in vertical SP (Sharpe, 2008)	Impaired adducting saccades (MLF lesion; slow velocity and reduced amplitude)	Fibers that make up the MLF: (1) AC and PC SCC (nystagmus), (2) utriculo-ocular motor (skew), (3) interneurons for conjugate horizontal movements (INO)	Foville syndrome -Contralateral hemiparesis and hemisensory loss. Ipsilateral 6th nerve palsy
			GEN: + (vertical [paramedian tracts])						
**Midbrain/superior cerebellar peduncle**	Basilar A, SCA, PCA	-	Spontaneous: + (vertical-torsional or pure torsional)	+ (ipsi hyper)	-	**INC**—vertical SP impaired **Midbrain tegmentum**—ipsilesional SP impaired **SCP**—horizontal bilateral smooth pursuit impairment	**riMLF** (impaired downward more than upward vertical saccades/loss of ipsi-torsional quick phases); **INC** (impaired vertical/torsional gaze holding)	Ocular motor abnormalities due to lesions involving 3rd nucleus/fascicle (ptosis, mydriasis, MR, SR, IR, IO paresis); 4^th^ nucleus/fascicle (SO); MLF; INC (vertical/torsional gaze holding); riMLF (vertical saccades/torsional quick phases)	3rd nerve palsy, vertical gaze palsy, one-and-a-half syndrome, contralateral weakness/sensory loss. 4th nerve palsy, contralateral INO, Horner's syndrome and/or ipsilateral hemi-ataxia, UB-torsional nystagmus
			GEN: + (vertical)						

Adapted from Source: Gold, D. R., Fracica, E. A., Hale, D., and Steenerson, K. (2023, February). Hints “plus” patterns in the acute vestibular syndrome based on location: Novel - Daniel Gold Collection. J. Willard Marriott Digital Library. https://collections.lib.utah.edu/ark:/87278/s6xrbk5t. AICA, anterior inferior cerebellar artery; contra, contralesional; GEN, gaze-evoked nystagmus; ASA, anterior spinal artery; MVN, medial vestibular nucleus; NPH, nucleus prepositus hypoglossi; HC, horizontal canal; VOR, vestibulo-ocular reflex; PICA, posterior inferior cerebellar artery; hyper, hypertropia; ipsi, ipsilesional; apogeo, apogeotropic; AN, periodic alternating nystagmus; HSN, head-shaking nystagmus; geo, geotropic; DBN, downbeat nystagmus; SCA, spinocerebellar ataxia; MLF, medial longitudinal fasciculus; AC, anterior canal; PC, posterior canal; SCC, semicircular canal; INO, internuclear ophthalmoplegia; SCA, superior cerebellar artery; PCA, posterior cerebral artery; MR, medial rectus; SR, superior rectus; IR, inferior rectus; IO, inferior oblique; SO, superior oblique; INC, interstitial nucleus of Cajal; riMLF, rostral interstitial medial longitudinal fasciculus. ++ often present. + can be present. ^−^absent. ^*^central HSN patterns.

#### 2.5.2. Flocculus and paraflocculus (tonsil)

The flocculus and the paraflocculus (tonsil) of the cerebellum play a key role in smooth pursuit, so lesions cause marked pursuit impairment (Shemesh and Zee, 2019). Unlike MCP strokes, unilateral lesions of the flocculus or the tonsil (paraflocculus) cause asymmetric impairments in smooth pursuit, being worse ipsilesionally. However, a lesion of the flocculus or paraflocculus rarely occurs in isolation. Floccular ischemia is commonly associated with AICA-territory stroke, which also irrigates the dorsolateral pons and ipsilateral labyrinth. In one reported case of isolated flocculus stroke, the patient had ipsilesional smooth pursuit impairment (Park et al., 2013). Patients with floccular ischemic stroke may also have skew, nystagmus (ipsilesional horizontal or horizontal–torsional), and a contralesional abnormal head impulse test (Brandt and Dieterich, 1993; Park et al., 2013; Yacovino et al., 2018; Shemesh and Zee, 2019).

Injury to the paraflocculus (tonsil) is more common with PICA-territory ischemia (Caplan and van Gijn, 2012). Although rarely occurring in isolation, there are case reports of an isolated paraflocculus ischemic stroke. Paraflocculus infarcts cause marked impairment in smooth pursuit (ipsilesional worse than contralesional) (Lee et al., 2014). Patients have also been found to have several patterns of nystagmus (including downbeat nystagmus with symmetric lesions and central pattern head-shaking nystagmus), ataxia, and intact vestibulo-ocular reflexes/head impulse test (Choi et al., 2018; Ogawa et al., 2018). Additional features of flocculus and paraflocculus strokes are summarized in Table 2.

Of note, when smooth pursuit is abnormal bilaterally, symmetrically, and in horizontal and vertical directions, a cerebellar (or its connections to/from the brainstem) disorder should be suspected. The presence of gaze-evoked nystagmus, downbeat nystagmus, and/or gait and limb ataxia makes localization to the cerebellum even more compelling.

### 2.6. Other stroke syndromes where abnormal smooth pursuit may occur

Other regions in the brainstem may cause abnormalities in smooth pursuit if infarcted, but smooth pursuit deficits are commonly overshadowed by other more significant neurologic deficits. These regions include the midbrain tegmentum (ipsilesional pursuit impairment); the dorsomedial midbrain affecting the interstitial nucleus of Cajal (INC; vertical pursuit impairment); the superior cerebellar peduncle (brachium conjunctivum), which is associated with horizontal bilateral pursuit impairment (Lee et al., 2015); the dorsomedial pons (ipsilesional pursuit impairment) (Thier et al., 1991); the lateral medulla (contralesional pursuit impairment) (Baloh et al., 1981); and the medial medulla (affecting the NPH-MVN) and therefore horizontal pursuit (Sharpe, 2008).

### 2.7. Summary

- Smooth pursuit function is dependent on higher cortical and subcortical structures involved in attention, alertness, and reward, such that assessment of smooth pursuit will be impacted by the patient's level of alertness/arousal, attention, and engagement.
- In peripheral causes of AVS, smooth pursuit function will be normal (except for a superimposed spontaneous unidirectional nystagmus following Alexander's law such that there will be a slow drift toward the paretic ear with a fast, corrective saccade away from the direction of the slow phase).
- Supratentorial causes of smooth pursuit impairment.
  ◦ Smooth pursuit and saccadic abnormalities often co-occur with supratentorial strokes, causing ipsilesional pursuit impairment and contralesional saccade abnormalities. They tend to be overshadowed by more significant neurologic signs and symptoms. Supratentorial strokes are not typically associated with dizziness, and these patients do not present with AVS.
- Infratentorial causes of smooth pursuit impairment.
  ◦ All the infratentorial stroke syndromes described here may present with acute onset continuous dizziness and may have a central pattern on the HINTS+ exam.
  ◦ Brainstem.
    ▪ With significant symmetric bilateral pursuit impairment in acute stroke, consider an MCP localization.
  ◦ Cerebellum.
    ▪ Strokes involving the cerebellar flocculus and the paraflocculus cause pursuit impairment ipsilesionally.
  ◦ An AICA-territory stroke may involve the dorsolateral pons, the MCP, and/or the flocculus, so smooth pursuit is often abnormal.

## 3. Saccades

### 3.1. Purpose

A saccade is a rapid, conjugate movement of the eyes to a visual target.

### 3.2. Assessing saccades

Saccades should be evaluated in terms of velocity, latency, accuracy, and conjugacy. One method to assess saccades is to have the patient looking alternatively between the examiner's nose and an eccentric target (approximately 15–20 degrees from the center) for both the horizontal and vertical directions. The eccentric fixation target should be something that is relatively small but can be seen clearly, for example, the tip of a reflex hammer or the examiner's fingertip (Normal Saccades VIDEO; Gold, 2016). Abnormalities in accuracy including hypometria (consistently undershooting the target) and hypermetria (consistently overshooting the target) will be apparent when the patient looks back at the nose. The patient should then make alternating saccades between two targets held apart at a greater amplitude, so-called self-paced saccades, while the examiner assesses saccade speed (too slow?), latency (too long?), and conjugacy (presence of an adduction lag?).

In cases where there is ocular motor weakness (either due to nerve palsy or muscle weakness), patients may have slow saccades and reduced saccadic amplitude in the direction of the weak muscle/nerve. Slow saccades without reduced amplitude can be seen in lesions affecting the horizontal and vertical burst neurons in the pons and midbrain, respectively (Slow Saccades VIDEO; Tourkevich and Gold, 2017). (NOVEL) Saccade assessment will be limited in the patient who is encephalopathic, delirious, lethargic, or even inattentive or uncooperative. In these instances, the examiner can assess the saccadic component or “fast phase” of optokinetic nystagmus (OKN), using an optokinetic drum or tape.

### 3.3. Anatomy of saccades

**Figure 2:**
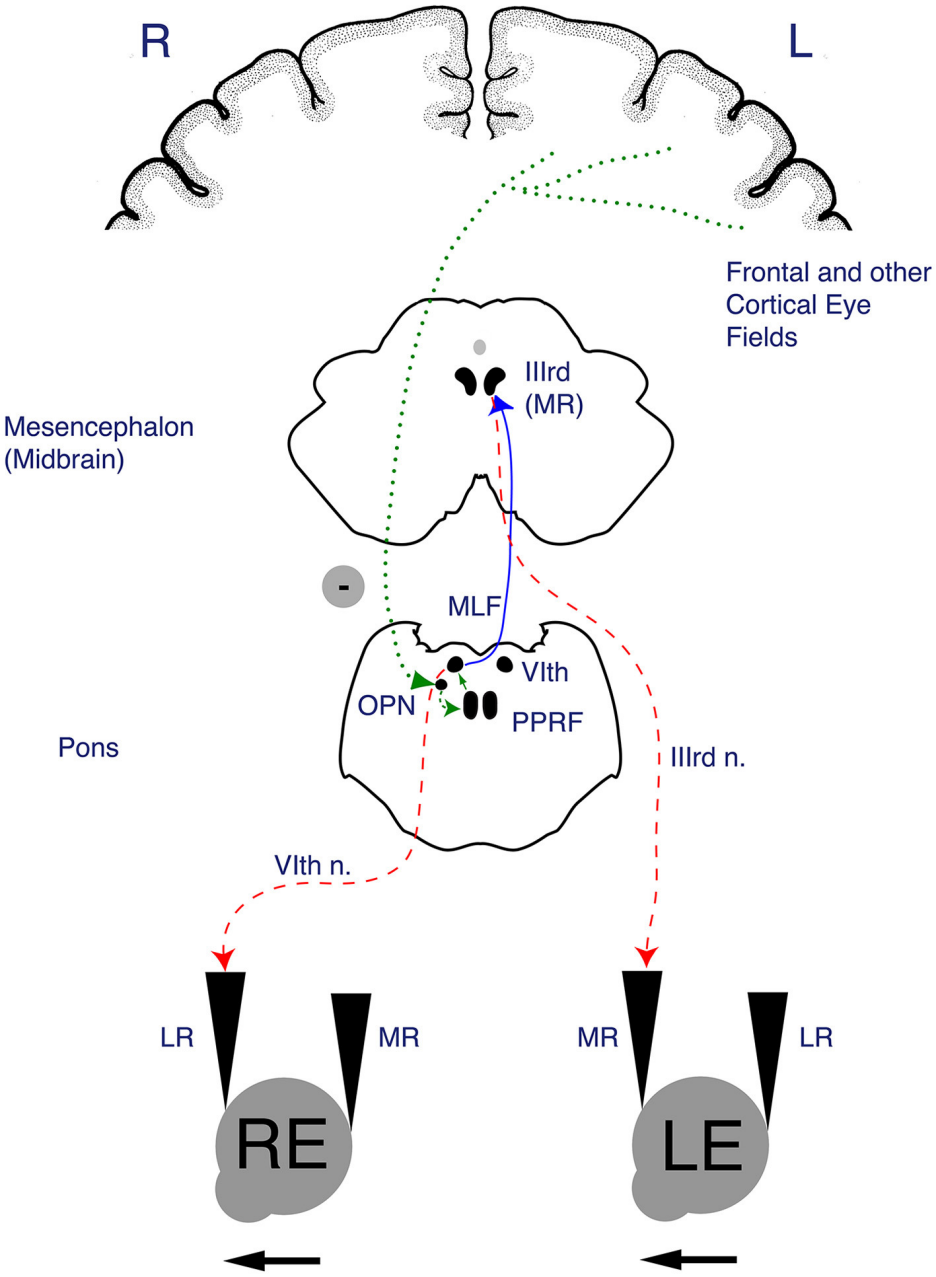
Anatomy of the horizontal saccade pathway. Once input from the afferent visual system (via the retina and optic tracts) reaches the frontal eye field (FEF) and parietal cortical eye fields, the descending fibers of the horizontal saccade pathway pass through subcortical structures including the basal ganglia, internal capsule and thalamus to the superior colliculus of the midbrain, which is involved in initiation of saccades. The R paramedian pontine reticular formation (PPRF) houses excitatory burst neurons that project to the R abducens nucleus. The R abducens nucleus sends fibers across the midline via the left medial longitudinal fasciculus (MLF_ to the left ocular motor nucleus (CN III). The end action of a horizontal saccade occurs when the R PPRF leads to a contraction of the R lateral rectus and the L medial rectus (via the MLF), thus facilitating a rightward horizontal saccade. Figure reproduced with permission from Liu, Volpe, and Galetta's Neuro-Ophthalmology: Diagnosis and Management. 3rd Edition. Copyright 2018, Elsevier.

As with smooth pursuit, a complex cortical network provides top-down modulation of the input from the primary visual pathway to affect the key brainstem centers for horizontal and vertical saccades. There are inputs from the FEF and parietal eye fields that pass through the basal ganglia and striatum on the way to the superior colliculus, playing a role in the initiation of saccades and target selection. Vertical saccades are initiated by the rostral interstitial medial longitudinal fasciculus (riMLF) in the midbrain. Horizontal saccades are initiated by the paramedian pontine reticular formation (PPRF) in the pons. The riMLF and the PPRF are referred to as excitatory burst neurons. Finally, the saccade command must be executed by the corresponding cranial nerve nuclei and extraocular muscles. This process is regulated by the DV and the fastigial oculomotor region (FOR) in the cerebellum. The anatomy of the horizontal saccade pathway is illustrated in Figure 2.

#### 3.3.1. Velocity and initiation

With lesions affecting the PPRF or the riMLF, the patient will demonstrate either reduced velocity saccades or an inability to initiate saccades. A unilateral PPRF lesion leads to slowed velocity or inability to generate ipsilesional horizontal saccades. A unilateral riMLF lesion leads to slowed velocity or difficulty initiating downward, greater than upward, saccades and absent ipsitorsional quick phases with head tilt.

#### 3.3.2. Accuracy

**Figure 3:**
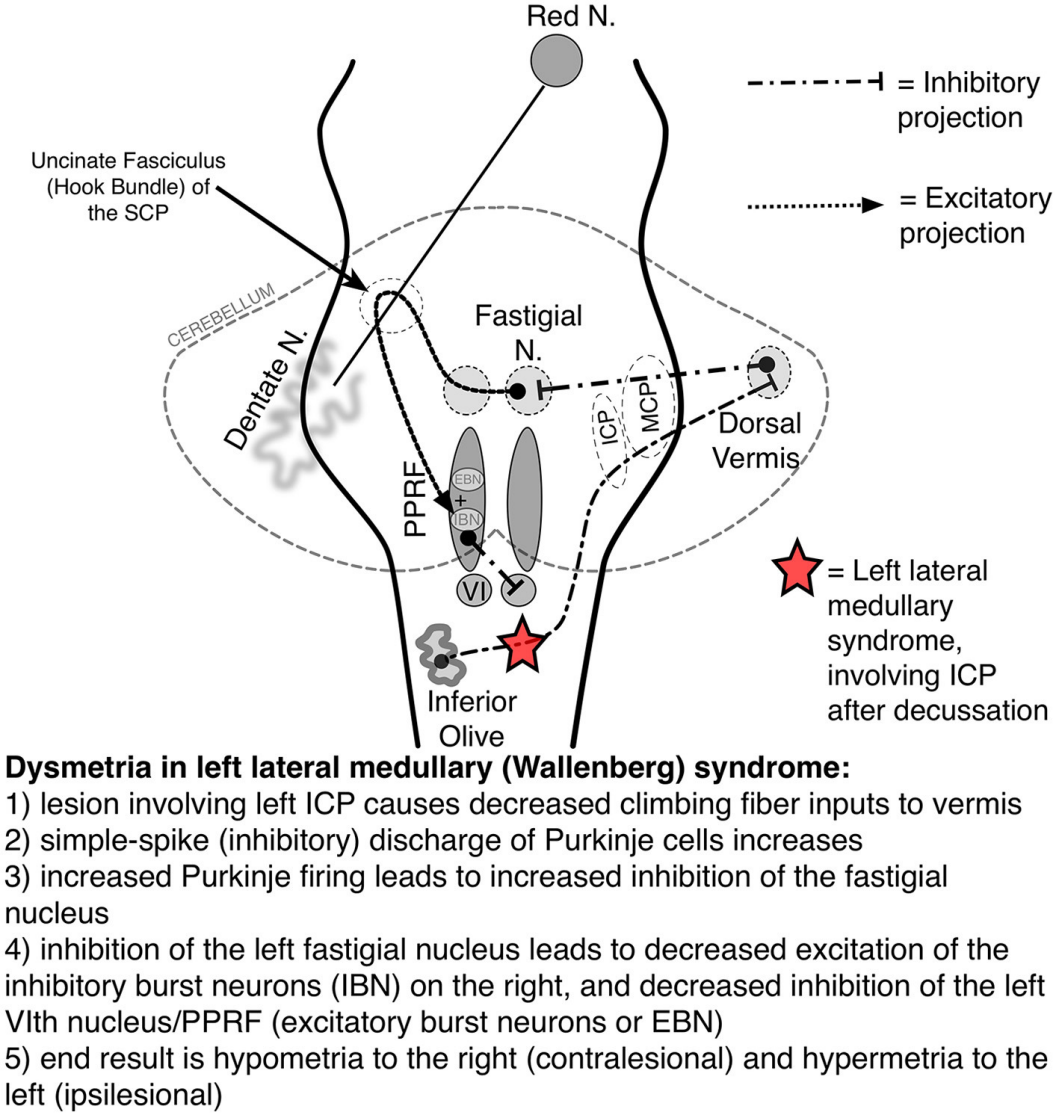
Saccadic pathways in the brainstem and cerebellum and mechanism for saccadic dysmetria in wallenberg syndrome—abnormal function of the brainstem/cerebellar saccadic pathways with a left wallenberg syndrome. Source: Neuro-Ophthalmology Virtual Education Library: NOVEL [Online]. Available online at: https://collections.lib.utah.edu/ark:/87278/s67h5cjg (accessed June 5, 2023). VI, sixth nerve nucleus; EBN, excitatory burst neuron; IBN, inhibitory burst neuron; ICP, inferior cerebellar peduncle; PPRF, paramedian pontine reticular formation; SCP, superior cerebellar peduncle.

Saccadic accuracy is mediated by projections from the FEF to pontine nuclei, which project to the DV and the FOR of the cerebellum before traveling rostrally again to the PPRF, which projects to the abducens nucleus to initiate horizontal saccades. The inferior olive and climbing fibers of the ICP, both medullary structures, also modulate cerebellar control of saccadic accuracy. Thus, injury to the supratentorial pathways, the cerebellum (DV and FOR), and/or medulla can produce impaired saccade accuracy (Figure 3) (Gold, 2017, 2021). Both unilateral and bilateral FOR lesions lead to bilateral hypermetria due to damage of bilaterally decussating fibers. In contrast, unilateral DV lesions produce ipsilateral hypometric saccades and contralateral hypermetric saccdes. Bilateral DV lesions produce bilaterally hypometric saccades (Shemesh and Zee, 2019; Gold, 2021).

#### 3.3.3. Conjugacy

Comparison of saccades in the two eyes can be helpful in identifying asymmetries between the two eyes. This is best observed when evaluating self-paced saccades, usually with saccades occurring slower in one eye when compared to the other. This pattern, for example, can be used to identify an internuclear ophthalmoplegia (INO) from a medial longitudinal fasciculus (MFL) lesion.

### 3.4. Anterior circulation strokes associated with abnormal saccades

**Vasculature**: MCA, ACA, ICA.

**Key Structures:** hemispheric, FEF, posterior parietal cortex.

In patients with supratentorial stroke, saccades are most commonly impaired contralesionally and may affect latency, accuracy, and/or velocity. As with smooth pursuit, numerous supratentorial regions can cause saccadic abnormalities, which have been described throughout the literature. Lesions of the FEF have been shown by Rivaud et al. to lead to decreased accuracy of contralateral saccades (Rivaud et al., 1994). Patients with lesions affecting the posterior parietal cortex have been shown to have an increase in saccade latency. Moreover, left posterior parietal cortex lesions have been shown to cause a contralateral increase in saccadic latency while right posterior parietal cortex lesions have been shown to increase saccade latency bilaterally, suggesting a greater involvement of the right parietal lobe in saccadic planning when compared to the left (Pierrot-Deseilligny et al., 1991). It has been shown by Braun et al. that damage to the dorsal parietal lobe leads to an increase in saccade latency, worse when the target was contralateral to the lesion (Braun et al., 1992). In a review of 47 patients with acute unilateral, supratentorial ischemic, and hemorrhagic strokes, patients more commonly had contralesional saccadic deficits than ipsilesional, specifically contralesional saccadic hypometria and reduced saccade latency and velocity were observed (Kudo et al., 2021). The authors found bedside video oculography assessment of the saccade function was a reliable and highly correlated biomarker with hemi-spatial neglect using the behavioral inattention test (Kudo et al., 2021).

### 3.5. Posterior circulation strokes associated with abnormal saccades

**Vasculature**: basilar perforators, PICA, ASA, vertebral arteries.

**Lateral medullary (Wallenberg) syndrome**.

In patients with AVS, the neurologist should recognize the lateral medullary (Wallenberg) stroke syndrome as having a characteristic pattern of saccadic dysmetria that can be highly localizing. This is especially helpful in subtle cases where patients lack other characteristic exam findings that could otherwise be used to localize symptoms. Specifically, a lateral medullary stroke causes *ipsilesional hypermetric saccades and contralesional hypometric saccades* in addition to the findings reviewed in Table 2. Upon eye closure, the eyes will drift ipsilesionally (ocular lateropulsion, also called ipsipulsion), requiring a series of hypometric (contralesional) saccades to re-center gaze once eyes are opened (Choi and Gold, 2019). The finding of ocular lateropulsion when present after brief eye closure (3–5 seconds) was found to be 100% specific for central etiology in one study looking at patients presenting with both peripheral and central causes of AVS (Kattah et al., 2020). There can also be a C-shaped arcing or bowing outward of the eyes toward the side of the lesion during vertical saccades (Kaski et al., 2012; Leigh and Zee, 2015).

Saccadic dysmetria and ipsipulsion occur in the Wallenberg syndrome because of injury to the medullary climbing fibers of the ICP, which provide input to the cerebellum (Figure 3) (Straube et al., 1994; Gold, 2017). In addition to regulating the accuracy of limb and truncal movements, the cerebellum plays a key role in the accuracy of saccadic eye movements and is connected with the pontine structures responsible for initiating the saccade. For example, as the right PPRF facilitates a rightward saccade, contralateral (leftward) saccades are inhibited via cerebellar pathways which end with inhibitory burst neurons (IBNs) at the pontomedullary junction (Figure 3).

Understanding the saccadic circuitry in the posterior fossa is essential to understanding why a particular lesion causes such a characteristic pattern of saccadic dysmetria. Take left Wallenberg syndrome as an example: beginning in the right inferior olive, medullary fibers decussate and become the left ICP climbing fibers, the region that is affected in Wallenberg syndrome (Figure 3) (Gold, 2017). The left ICP fibers normally inhibit the Purkinje cells of the cerebellar DV, which, in turn, inhibits the left FN of the cerebellum. The FN excites the contralateral EBNs (facilitating contralateral saccades) but the left FN also projects to the ipsilateral IBNs (to inhibit ipsilateral saccades). Thus, left Wallenberg syndrome results in decreased contralateral (rightward) saccadic tone and relatively increased ipsilateral (leftward) saccadic tone. This causes the findings of ipsipulsion (leftward), ipsilesional (left) hypermetria, and contralesional (right) hypometria (VIDEO; Gold, 2016).

Lateral medullary syndrome is most commonly due to vertebral or PICA occlusion (Fisher et al., 1961). Several studies have found vertebral artery disease is more commonly observed than disease affecting the PICA among patients with lateral medullary strokes (Norrving and Cronqvist, 1991; Sacco, 1993; Kim, 2003).

### 3.6. Other vestibular strokes where saccadic abnormalities may be observed

**Key Structures:** MLF +/– PPRF (horizontal), medial midbrain (INC + riMLF) (vertical saccades), medial medulla, and cerebellum.

We will review other regions supplied by the posterior circulation, which may cause saccadic abnormalities.

#### 3.6.1. Medial pons

##### 3.6.1.1. MLF syndrome (horizontal saccadic conjugacy)

A lesion affecting the MLF often leads to a motility deficit affecting the ipsilateral medial rectus with an adduction deficit (INO), with an associated abducting nystagmus in the contralateral eye (Lee et al., 2022). In milder cases, patients may only demonstrate slowed adducting saccades, so-called adduction lag. For example, when assessing self-paced saccades in a patient with a right MLF lesion, the right eye adducting saccades will be slower in comparison to the left eye abducting saccades. In addition to INO, MLF strokes often cause vertigo and acute spontaneous dissociated vertical-torsional nystagmus due to the involvement of the vertical semicircular canal fibers that travel through the MLF (Lee et al., 2022). MLF lesions can also result in the pathological ocular tilt reaction, a triad of skew deviation (ipsilateral hypertrophia), contralateral head tilt, and ocular counter-roll due to damage of the utriculo-ocular motor pathways, which also travel through the MLF (Gold, 2021).

##### 3.6.1.2. MLF and one-and-a-half syndrome (MLF + abducens nucleus)

The abducens nucleus contains not only the neurons that will innervate the ipsilateral abducens nerve (and lateral rectus) but also the interneurons that will innervate the contralateral medial rectus to allow for conjugate horizontal movements. Therefore, a unilateral (right) abducens nucleus lesion leads to an ipsilateral (rightward) horizontal gaze palsy, involving ipsilateral saccades, smooth pursuit, and horizontal VOR. The abducens nucleus is proximal to the MLF such that both may be affected ipsilaterally in the setting of a stroke. The resulting syndrome is often termed a “one-and-a-half syndrome,” so that a right pontine lesion can cause a rightward gaze palsy and right INO, leaving only contralateral (left) abduction intact (Gold, 2021).

##### 3.6.1.3. PPRF vs. abducens nucleus in horizontal conjugate gaze palsy

Pure PPRF lesions causing an isolated horizontal (ipsilesonal) saccadic palsy are rare, and there are important differences between PPRF and abducens nucleus lesions clinically. A lesion of the PPRF, which contains the horizontal excitatory burst neurons, results in the inability to generate or slow ipsilateral saccades. However, these patients will still have intact smooth pursuit and VOR due to an intact abducens nucleus (Gold, 2021). While PPRF lesions in isolation would not be expected to cause vertigo, given its proximity to the vertical semicircular canal and utriculo-ocular motor pathways (e.g., MLF and ventral tegmental tract), vestibular symptoms may be associated. PPRF lesions may occur with MLF lesions due to their proximity.

#### 3.6.2. Medial medulla

A medial medullary stroke (including Dejerine syndrome) is far less common than lateral medullary syndrome, representing <1% of PCSs (Bassetti et al., 1997). While rarely reported, Kim et al. (2004) report a case where a patient with a unilateral medial medullary lesion had ipsilesional saccadic hypometria and contralesional hypermetric saccades (i.e., the opposite pattern from what is observed in a lateral medullary infarct). This is thought to occur in the setting of injury to the saccade pathway fibers from the inferior olive prior to decussation (whereas Wallenberg involves hitting these fibers post-decussation). Ocular lateropulsion with eyes drifting contralesionally (or contrapulsion) may also be observed in medial medullary stroke (Gold, 2021).

Kameda et al. (2004) compared cases of lateral and medial medullary infarction and found vertebral artery dissection is an important cause of both lateral and medial medullary strokes, representing 29 and 21% of cases, respectively. Medial medullary strokes are most commonly due to vertebral disease or atherosclerotic disease at the vertebro-basilar junction, but may also occur due to anterior spinal artery stroke, especially when seen in conjunction with a rostral spinal cord infarct or when located in the caudal medial medulla (Caplan and van Gijn, 2012).

#### 3.6.3. Medial midbrain (riMLF, INC, posterior commissure; vertical saccades)

As a short heuristic, while the pons is important for horizontal eye movements and saccades, the midbrain is important for vertical eye movements, especially gaze-holding and saccades. In specifically considering locations that can lead to vertigo and saccadic abnormalities, the INC would be most likely to co-occur with vertigo. We will briefly mention the riMLF, which in isolation should not cause dizziness but is responsible for vertical saccades. The posterior commissure (PC) also does not typically cause vertigo, lesions here (especially as part of dorsal midbrain syndrome) cause highly localizing neuro-ophthalmic and ocular motor signs that are important for the stroke neurologist to recognize in the context of patients presenting with hydrocephalus in the setting of large ischemic or hemorrhagic strokes and resultant hydrocephalus and herniation. Dieterich et al. found that midbrain lesions were associated with rotational vertigo in 14% of cases, typically lasting <1 day, and among those with midbrain tegmentum or meso-diencephalic lesions, 22% experienced swaying or unspecific dizziness, and 33% endorsed postural imbalance.

##### 3.6.3.1. Interstitial nucleus of cajal

The INC is the neural integrator for vertical and torsional gaze-holding. In primary gaze, there are no orbital elastic forces to overcome, so the extraocular muscles are relatively relaxed. To make a saccade to the left and then tonically hold the eyes in a far-left gaze, there is an initial saccadic *pulse* command, where the excitatory burst neurons lead to a large pulse activation of the agonist muscles and the IBNs provide a lesser command to relax the antagonist muscles, thus facilitating the initial leftward burst of movement in a saccade. To hold the eyes in a leftward gaze once the saccade has been made (against the elastic forces that would otherwise cause the eye to drift back to center), there is a *step* command that allows for a steady eccentric gaze without centripetal drift. The step command is generated by the neural integrator (which integrates the eye velocity to an eye position command). The INC is the neural integrator for vertical and torsional eye movements (Sadeghpour et al., 2019).

In a unilateral (right) INC lesion, vertical saccades and smooth pursuit will be intact, but there can be vertical gaze-evoked nystagmus and spontaneous torsional (ipsiversive, toward the right ear) nystagmus. There will also be a contraversive (leftward) ocular tilt reaction, with an ipsilesional (right) hypertropia. In bilateral INC lesions, all vertical eye movements (upgaze and downgaze) including saccades, smooth pursuit, and VOR may be impaired, with associated vertical gaze-evoked nystagmus (Strupp et al., 2014).

##### 3.6.3.2. Rostral interstitial MLF

The EBNs for vertical saccades are found in the rostral interstitial nucleus of the riMLF, which is located at the rostral pole of the red nucleus (Caplan and van Gijn, 2012; Leigh and Zee, 2015). A unilateral (right) riMLF lesion will cause slowing of downward more than upward saccades without affecting smooth pursuit, and cause a spontaneous torsional (contraversive, toward the left ear) nystagmus (Leigh and Zee, 2015). There is also a loss of ipsitorsional (rightward) quick phases, which would normally be observed when tilting the head ipsilesionally as the riMLF contains burst neurons for not only vertical saccades but also for torsional quick phases. In bilateral riMLF lesions, vertical saccades upward and downward can be severely impaired or abolished but with intact smooth pursuit, VOR, and vertical gaze-holding (Leigh and Zee, 2015; Gold, 2021).

The riMLF is typically supplied by the posterior thalamo-subthalamic paramedian artery (superior ramus of P1). Additional supplied structures include the subthalamus, red nucleus, and posterior-inferior portions of the thalamus. In bilateral riMLF infarcts, the artery of Percheron should be suspected (Caplan and van Gijn, 2012; Leigh and Zee, 2015). Diencephalic lesions interrupting descending riMLF inputs can lead to selective palsy of voluntary vertical saccades with intact upward and downward fast phases on OKN testing (Leigh and Zee, 2015). Parinaud's syndrome and lesions affecting the PC may also occur with the “top-of-the-basilar” syndrome. Posterior commissure may be infarcted with superior cerebellar artery occlusion or with paramedian thalamic artery occlusion (Caplan and van Gijn, 2012).

### 3.7. Summary

- Saccades should be evaluated using the following criteria: accuracy, velocity, latency, and conjugacy in both horizontal and vertical planes.
- In peripheral causes of AVS, saccades will be normal (with the exception of a superimposed spontaneous unidirectional nystagmus following Alexander's law such that there will be a slow drift toward the paretic ear with fast, corrective saccades away from the direction of the slow phase).
- Supratentorial causes of saccade abnormalities.
  ◦ Remember, supratentorial strokes are not typically associated with dizziness, and these patients do not present with AVS.
  ◦ Smooth pursuit and saccadic abnormalities often co-occur, especially with supratentorial lesions. Saccades are typically impaired contralesionally.
  ◦ Anterior circulation causes of saccade abnormalities will tend to result in more diffuse problems with multiple aspects of saccade performance.
- Infratentorial causes of saccade abnormalities.
  ◦ Saccadic abnormalities provide more localizing value in the brainstem and cerebellum.
  ◦ The riMLF and PPRF burst neurons affect the velocity and initiation of saccades in vertical and horizontal directions, respectively.
  ◦ Saccadic hypermetria is common with cerebellar (or its connections) disorders, while saccadic hypometria may be seen with cerebral, basal ganglia (e.g., parkinsonism), and/or cerebellar (ocular motor vermis) disorders. Strokes affecting the cerebellum can acutely present with hypermetric or hypometric saccades.
  ◦ The pattern of ipsipulsion, ipsilesional hypermetria, and contralesional hypometria is highly localizing in the Wallenberg syndrome.
  ◦ Among the infratentorial saccadic abnormalities summarized here, a lateral medullary stroke (Wallenberg) or a cerebellar stroke will commonly present with AVS and acute onset continuous dizziness. While a tiny isolated PPRF or riMLF stroke would not be expected to cause dizziness, the neighboring structures that are commonly affected during a stroke in this region can cause dizziness and an abnormal central HINTS+ exam.

## 4. Conclusion

The HINTS+ exam is the gold-standard clinical bedside test that plays a critical role in evaluating patients with AVS. While the HINTS+ exam is highly sensitive and specific when performed and interpreted correctly, its performance is less reliable in the hands of non-specialists.

Provider experience and comfort level may be associated with the risk of misinterpreting findings (e.g., when the head impulse test is performed too gingerly, such that the examiner fails to elicit a catch-up saccade despite a peripheral deficit). Sometimes in AVS due to a stroke, the nystagmus is so subtle that it is only observed with fixation removal, which not all providers may be comfortable performing.

In cases where a patient with AVS has a clear central pattern HINTS+ exam, the diagnosis is clear. It is in these more subtle presentations and when the HINTS+ exam is performed by non-experts that additional clinical exam findings may be helpful. In particular, incorporating the assessment of saccades and smooth pursuit in addition to the HINTS+ exam may help neurologists “localize the lesion.” The anatomy of the saccade and smooth pursuit pathways is complex, but we hope that this clinically oriented review of the pattern of deficits expected with strokes affecting different structures in each pathway from the cortex to the medulla will lead to the translation and adoption of this knowledge at the bedside.

In particular, there are a few specific infratentorial stroke syndromes where saccade and smooth pursuit testing can help with localizing the lesion to the brainstem or cerebellum. In most of these cases, patients can present with AVS either due to direct injury to the structures in these pathways or because neighboring structures that are also commonly involved can cause AVS. The key brainstem vestibular stroke syndromes that cause abnormal horizontal smooth pursuit include lesions of the MCP and dorsolateral pons (both supplied by AICA) and the cerebellar flocculus and the paraflocculus (tonsil; supplied by PICA). Unilateral lesions of the MCP impair smooth pursuit bilaterally, while unilateral cerebellar lesions impair smooth pursuit ipsilesionally.

The key brainstem vestibular stroke syndromes affecting saccades are MLF syndrome (when there is an adduction lag or paresis in the ipsilateral eye) and lateral medullary (Wallenberg) syndrome, where there is ipsipulsion, ipsilesional hypermetria, and contralesional hypometria. A medial pontine stroke affecting the PPRF causes a distinctive ipsilesional horizontal saccadic palsy and may present with AVS and central HINTS+ when adjacent vestibular structures are affected. A medial midbrain stroke affecting the riMLF typically will not present with dizziness, but the characteristic impairment of vertical saccades (affecting downward, more than upward, saccades) is critical to recognize as a sign of brainstem ischemia and can be seen with the “top of the basilar” syndrome.

Although the HINTS+ exam should be relied on in the AVS, associated pursuit and saccadic abnormalities can increase the examiner's confidence in a central disorder when there is uncertainty and when confounding features are present (e.g., patient is blind in one eye so a test of skew cannot be performed; patient has a preexisting unilateral vestibular loss due to vestibular schwannoma so head impulse test is challenging to interpret; or patient has a baseline mild gaze-evoked nystagmus due to anti-seizure medication, so the pattern of nystagmus cannot be relied on). Evaluating pursuit and saccades along with the HINTS+ exam is rapid, easy to perform (even for the non-subspecialist), and effective (when abnormalities are interpreted correctly), so we feel that they are, in fact, worthwhile, and we would advocate their routine assessment in the AVS.

We hope that by reviewing how to assess and interpret these additional components of the ocular motor exam, readers will have an additional tool they can use to complement the HINTS+ exam, especially in cases where there is diagnostic uncertainty. The implementation of this knowledge at the bedside can help examiners more accurately localize central deficits, as well as provide insight into possible etiologies and/or vascular territories involved.

## Author contributions

EF and DG contributed to conception and design of the manuscript. EF wrote the first draft of the manuscript. DH wrote sections of the manuscript. All authors contributed to manuscript revision, read, and approved the submitted version.

## References

[B1] Baloh R. W. Yee R. D. Honrubia V. (1981). Eye movements in patients with wallenberg's syndrome. Ann. N Y. Acad. Sci. 374, 600–613. 10.1111/j.1749-6632.1981.tb30904.x 6978648

[B2] Bassetti C. Bogousslavsky J. Mattle H. Bernasconi A. (1997). Medial medullary stroke: Report of seven patients and review of the literature. Neurology. 48, 882–890. 10.1212/WNL.48.4.882 9109872

[B3] Bisdorff A. R. Staab J. P. Newman-Toker D. E. (2015). Overview of the international classification of vestibular disorders. Neurol. Clin. 33, 541–550. 10.1016/j.ncl.2015.04.010 26231270

[B4] Brandt T. Dieterich M. (1993). Skew deviation with ocular torsion: A vestibular brainstem sign of topographic diagnostic value. Ann. Neurol. 33, 528–534. 10.1002/ana.410330518 8498829

[B5] Braun D. Weber H. Mergner T. Schulte-Mönting J. (1992). Saccadic reaction times in patients with frontal and parietal lesions. Brain. 115, 1359–1386. 10.1093/brain/115.5.1359 1422793

[B6] Caplan L. van Gijn J. (2012). Stroke Syndromes. 3rd ed. New York: Cambridge University Press. 621. 10.1017/CBO9781139093286

[B7] Carmona S. Martínez C. Zalazar G. Moro M. Batuecas-Caletrio A. Luis L.. (2016). The diagnostic accuracy of truncal ataxia and HINTS as cardinal signs for acute vestibular syndrome. Front. Neurol. 7, 125. 10.3389/fneur.2016.00125 27551274 PMC4976483

[B8] Chang T. P. Zee D. S. Gold D. R. (2021). Upbeat nystagmus in dorsolateral pontine infarction. J. Neuroophthalmol. 41, e94–e96. 10.1097/WNO.0000000000000928 32141978

[B9] Choi S. Y. Jang J. Y. Oh E. H. Choi J. H. Park J. Y. Lee S. H.. (2018). Persistent geotropic positional nystagmus in unilateral cerebellar lesions. Neurology. 91, e1053–e1057. 10.1212/WNL.0000000000006167 30097474

[B10] Choi W. Y. Gold D. R. (2019). Ocular motor and vestibular disorders in brainstem disease. J. Clin. Neurophysiol. 36, 396–404. 10.1097/WNP.0000000000000593 31688322

[B11] Deplanque D. Godefroy O. Guerouaou D. Laureau E. Desaulty A. (1998). Sudden bilateral deafness: lateral inferior pontine infarction. J. Neurol. Neurosurg. Psychiatry. 64, 817a−8a. 10.1136/jnnp.64.6.817a 9647322 PMC2170138

[B12] Fisher C. M. Karnes W. E. Kubik C. S. (1961). Lateral medullary infarction—the pattern of vascular occlusion. J. Neuropath. Exp. Neurol. 20, 323–379. 10.1097/00005072-196107000-00001 13699936

[B13] FitzGibbon E. J. Calvert P. C. Dieterich M. Brandt T. Zee D. S. (1996). Torsional nystagmus during vertical pursuit. J. Neuro-Ophthalmol. 16, 79–90. 10.1097/00041327-199606000-00001 8797162

[B14] Gaymard B. Pierrot-Deseilligny C. Rivaud S. Velut S. (1993). Smooth pursuit eye movement deficits after pontine nuclei lesions in humans. J. Neurol. Neurosurg. Psychiatry. 56, 799–807. 10.1136/jnnp.56.7.799 8331357 PMC1015063

[B15] Gold D. R. (2016). Saccadic dysmetria and ocular lateropulsion in lateral medullary stroke. Spencer S. Eccles Health Sciences Library, University of Utah, 10 N 1900 E SLC, UT 84112-5890. Available online at: https://collections.lib.utah.edu/ark:/87278/s65176w6 (accessed June 18, 2023).

[B16] Gold D. R. (2017). Saccadic Pathways in the Brainstem and Cerebellum and Mechanism for Saccadic Dysmetria in Wallenberg Syndrome - Abnormal Function of the Brainstem/Cerebellar Saccadic Pathways with a Left Wallenberg Syndrome. Spencer S. Eccles Health Sciences Library, University of Utah, 10 N 1900 E SLC, UT 84112-5890. Available online at: https://collections.lib.utah.edu/ark:/87278/s67h5cjg (accessed June 18, 2023).

[B17] Gold D. R. (2018). Impaired Smooth Pursuit and Other Characteristic Ocular Motor Findings in Middle Cerebellar Peduncle Stroke. Spencer S. Eccles Health Sciences Library, University of Utah, 10 N 1900 E SLC, UT 84112-5890. Available online at: https://collections.lib.utah.edu/details?id=1307533 (accessed June 18, 2023).

[B18] Gold D. R. (2021). Neuro-Ophthalmology and Neuro-Otology: A Case-Based Guide for Clinicians and Scientists. Cham: Springer Nature. 10.1007/978-3-030-76875-1

[B19] Heide W. Kurzidim K. Kömpf D. (1996). Deficits of smooth pursuit eye movements after frontal and parietal lesions. Brain. 119, 1951–1969. 10.1093/brain/119.6.1951 9010000

[B20] Kameda W. Kawanami T. Kurita K. Daimon M. Kayama T. Hosoya T.. (2004). Lateral and medial medullary infarction: a comparative analysis of 214 patients. Stroke. 35, 694–699. 10.1161/01.STR.0000117570.41153.35 14963274

[B21] Kaski D. Bentley P. Lane R. Bronstein A. (2012). Up-down asymmetry of saccadic contrapulsion in lateral medullary syndrome. J. Neuroophthalmol. 32, 224–226. 10.1097/WNO.0b013e3182606bcd 22790667

[B22] Kato I. Takeyama I. Watanabe J. Nakamura T. Harada K. Hasegawa Y.. (1991). EOG findings in patients with lesions in cerebellar peduncles. Acta Otolaryngol. 111, 260–261. 10.3109/00016489109131396 1927389

[B23] Kattah J. C. Badihian S. Pula J. H. Tarnutzer A. A. Newman-Toker D. E. Zee D. S.. (2020). Ocular lateral deviation with brief removal of visual fixation differentiates central from peripheral vestibular syndrome. J. Neurol. 267, 3763–3772. 10.1007/s00415-020-10100-5 32719976 PMC9106094

[B24] Kattah J. C. Martinez C. Zalazar G. Batuecas Á. Lemos J. Carmona S.. (2022). Role of incubitus truncal ataxia, and equivalent standing grade 3 ataxia in the diagnosis of central acute vestibular syndrome. J. Neurol. Sci. 441, 120374. 10.1016/j.jns.2022.120374 36063733

[B25] Kattah J. C. Talkad A. V. Wang D. Z. Hsieh Y. H. Newman-Toker D. E. (2009). HINTS to diagnose stroke in the acute vestibular syndrome: three-step bedside oculomotor examination more sensitive than early mri diffusion-weighted imaging. Stroke. 40, 3504–3510. 10.1161/STROKEAHA.109.551234 19762709 PMC4593511

[B26] Kerber K. A. Brown D. L. Lisabeth L. D. Smith M. A. Morgenstern L. B. (2006). Stroke among patients with dizziness, vertigo, and imbalance in the emergency department: a population-based study. Stroke. 37, 2484–2487. 10.1161/01.STR.0000240329.48263.0d 16946161 PMC1779945

[B27] Kim H. A. Lee H. (2010). Isolated vestibular nucleus infarction mimicking acute peripheral vestibulopathy. Stroke. 41, 1558–1560. 10.1161/STROKEAHA.110.582783 20489171

[B28] Kim J. S. (2003). Pure lateral medullary infarction: clinical-radiological correlation of 130 acute, consecutive patients. Brain. 126, 1864–1872. 10.1093/brain/awg169 12805095

[B29] Kim J. S. Moon S. Y. Kim K. Y. Kim H. C. Park S. H. Yoon B. W.. (2004). Ocular contrapulsion in rostral medial medullary infarction. Neurology. 63, 1325–1327. 10.1212/01.WNL.0000140704.83719.B9 15477568

[B30] Kim M. Park S. Y. Lee S. E. Lee J. S. Hong J. M. Lee S. J.. (2022). Significance of vertigo, imbalance, and other minor symptoms in hyperacute treatment of posterior circulation stroke. Front. Neurol. 13, 845707. 10.3389/fneur.2022.845707 35651338 PMC9150563

[B31] Kim S. H. Kim H. J. Kim J. S. (2017). Isolated vestibular syndromes due to brainstem and cerebellar lesions. J. Neurol. 264, 63–69. 10.1007/s00415-017-8455-6 28314977

[B32] Kim S. H. Kim J. S. (2019). Eye movement abnormalities in middle cerebellar peduncle strokes. Acta Neurol. Belg. 119, 37–45. 10.1007/s13760-017-0860-1 29129037

[B33] Kudo Y. Takahashi K. Sugawara E. Nakamizo T. Kuroki M. Higashiyama Y.. (2021). Bedside video-oculographic evaluation of eye movements in acute supratentorial stroke patients: A potential biomarker for hemispatial neglect. J. Neurol. Sci. 425, 117442. 10.1016/j.jns.2021.117442 33857735

[B34] Lee S. H. Kim J. M. Kim J. S. (2022). Update on the medial longitudinal fasciculus syndrome. Neurol. Sci. 43, 3533–3540. 10.1007/s10072-022-05967-3 35258687

[B35] Lee S. H. Park S. H. Kim J. S. Kim H. J. Yunusov F. Zee D. S.. (2014). Isolated unilateral infarction of the cerebellar tonsil: Ocular motor findings: Tonsillar Infarction. Ann. Neurol. 75, 429–434. 10.1002/ana.24094 24812698

[B36] Lee S. U. Bae H. J. Kim J. S. (2015). Ipsilesional limb ataxia and truncal ipsipulsion in isolated infarction of the superior cerebellar peduncle. J. Neurol. Sci. 349, 251–253. 10.1016/j.jns.2015.01.006 25592415

[B37] Leigh R. J. Zee D. S. (2015). The Neurology of Eye Movements. 5th ed. Oxford University Press. Available online at: http://www.oxfordmedicine.com/view/10.1093/med/9780199969289.001.0001/med-9780199969289 (accessed May 8, 2022).

[B38] Lekwuwa G. U. Barnes G. R. (1996). Cerebral control of eye movements: I. The relationship between cerebral lesion sites and smooth pursuit deficits. Brain. 119, 473–490. 10.1093/brain/119.2.473 8800943

[B39] Machner B. Choi J. H. Neumann A. Trillenberg P. Helmchen C. (2021). What guides decision-making on intravenous thrombolysis in acute vestibular syndrome and suspected ischemic stroke in the posterior circulation? J. Neurol. 268, 249–264. 10.1007/s00415-020-10134-9 32772173 PMC7815559

[B40] Merwick A. Werring D. (2014). Posterior circulation ischaemic stroke. BMJ. 348, g3175–g3175. 10.1136/bmj.g3175 24842277

[B41] Morrow M. J. Sharpe J. A. (1990). Cerebral hemispheric localization of smooth pursuit asymmetry. Neurology. 40, 284–284. 10.1212/WNL.40.2.284 2300251

[B42] Newman-Toker D. E. (2016). Missed stroke in acute vertigo and dizziness: It is time for action, not debate: Missed Stroke in Acute Vertigo. Ann. Neurol. 79, 27–31. 10.1002/ana.24532 26418192 PMC9041814

[B43] Newman-Toker D. E. Hsieh Y. H. Camargo C. A. Pelletier A. J. Butchy G. T. Edlow J. A.. (2008). Spectrum of dizziness visits to us emergency departments: cross-sectional analysis from a nationally representative sample. Mayo. Clin. Proc. 83, 765–775. 10.4065/83.7.765 18613993 PMC3536475

[B44] Newman-Toker D. E. Peterson S. M. Badihian S. Hassoon A. Nassery N. Parizadeh D.. (2022). Diagnostic errors in the emergency department: a systematic review. agency for healthcare research. and quality (AHRQ). Available online at: https://effectivehealthcare.ahrq.gov/products/diagnostic-errors-emergency/research 36574484

[B45] Norrving B. Cronqvist S. (1991). Lateral medullary infarction: Prognosis in an unselected series. Neurology. 41:244–244. 10.1212/WNL.41.2_Part_1.244 1992369

[B46] Ogawa K. Suzuki Y. Akimoto T. Morita A. Hara M. Yoshihashi H.. (2018). Clinical study on 3 patients with infarction of the vermis/tonsil in the cerebellum. J. Stroke Cerebrovasc. Dis. 27, 2919–2925. 10.1016/j.jstrokecerebrovasdis.2018.05.040 30122628

[B47] Ohle R. Montpellier R. Marchadier V. Wharton A. McIsaac S. Anderson M.. (2020). Can emergency physicians accurately rule out a central cause of vertigo using the HINTS examination? A systematic review and meta-analysis. Acad. Emerg. Med. 27, 887–896. 10.1111/acem.13960 32167642

[B48] Park H. K. Kim J. S. Strupp M. Zee D. S. (2013). Isolated floccular infarction: impaired vestibular responses to horizontal head impulse. J. Neurol. 260, 1576–1582. 10.1007/s00415-013-6837-y 23370610

[B49] Park J. Y. Choi J. H. Kwon J. H. Weon Y. C. Lee S. M. Kim H. J.. (2023). Incidence, characteristics, and neuroanatomical substrates of vestibular symptoms in supratentorial stroke. J. Neurol. 270, 2174–2183. 10.1007/s00415-023-11566-9 36633670

[B50] Paul N. L. Simoni M. Rothwell P. M. (2013). Transient isolated brainstem symptoms preceding posterior circulation stroke: a population-based study. Lancet Neurol. 12, 65–71. 10.1016/S1474-4422(12)70299-5 23206553 PMC3530272

[B51] Pierrot-Deseilligny C. Rivaud S. Gaymard B. Agid Y. (1991). Cortical control of reflexive visually-guided saccades. Brain. 114, 1473–1485. 10.1093/brain/114.3.1473 2065261

[B52] Rivaud S. Mri R. M. Gaymard B. Vermersch A. I. Pierrot-Deseilligny C. (1994). Eye movement disorders after frontal eye field lesions in humans. Exp. Brain Res. 102, 232443. 10.1007/BF00232443 7895787

[B53] Sacco R. L. (1993). Wallenberg's lateral medullary syndrome: clinical-magnetic resonance imaging correlations. Arch. Neurol. 50, 609. 10.1001/archneur.1993.00540060049016 8503798

[B54] Sadeghpour S. Zee D. S. Leigh R. J. (2019). “Clinical applications of control systems models: The neural integrators for eye movements,” in Progress in Brain Research (Elsevier) 103–14. Available online at: https://linkinghub.elsevier.com/retrieve/pii/S0079612318302310 (accessed June 23, 2023). 10.1016/bs.pbr.2018.12.001 31239124

[B55] Searls D. E. (2012). Symptoms and signs of posterior circulation ischemia in the new england medical center posterior circulation registry. Arch. Neurol. 69, 346. 10.1001/archneurol.2011.2083 22083796

[B56] Sharpe J. A. (2008). Neurophysiology and neuroanatomy of smooth pursuit: Lesion studies. Brain Cogn. 68, 241–254. 10.1016/j.bandc.2008.08.015 19004537

[B57] Sharpe J. A. Morrow M. J. (1991). Cerebral hemispheric smooth pursuit disorders. Acta Neurol Belg. 91, 81–96. 10.3109/01658109108997300 2063647

[B58] Shemesh A. A. Zee D. S. (2019). Eye movement disorders and the cerebellum. J. Clin. Neurophysiol. 36, 405–414. 10.1097/WNP.0000000000000579 31688323 PMC6986321

[B59] Straube A. Helmchen C. Robinson F. Fuchs A. Büttner U. (1994). Saccadic dysmetria is similar in patients with a lateral medullary lesion and in monkeys with a lesion of the deep cerebellar nucleus. J. Vestib. Res. Equilib. Orientat. 4, 327–333. 10.3233/VES-1994-4502 7994478

[B60] Strupp M. Kremmyda O. Adamczyk C. Böttcher N. Muth C. Yip C. W.. (2014). Central ocular motor disorders, including gaze palsy and nystagmus. J. Neurol. 261, 542–558. 10.1007/s00415-014-7385-9 25145891 PMC4141156

[B61] Tarnutzer A. A. Lee S. H. Robinson K. A. Wang Z. Edlow J. A. Newman-Toker D. E.. (2017). ED misdiagnosis of cerebrovascular events in the era of modern neuroimaging: A meta-analysis. Neurology. 88, 1468–1477. 10.1212/WNL.0000000000003814 28356464 PMC5386439

[B62] Thier P. Bachor A. Faiss J. Dichgans J. Koenig E. (1991). Selective impairment of smooth-pursuit eye movements due to an ischemic lesion of the basal pons. Ann. Neurol. 29, 443–448. 10.1002/ana.410290419 1929215

[B63] Tourkevich R. Gold D. R. (2017). Ocular Motor Signs in Early Progressive Supranuclear Palsy. Spencer S. Eccles Health Sciences Library, University of Utah, 10 N 1900 E SLC, UT 84112-5890. Available online at: https://collections.lib.utah.edu/ark:/87278/s6gr0vxw (accessed June 18, 2023).

[B64] Tsao C. W. Aday A. W. Almarzooq Z. I. Anderson C. A. M. Arora P. Avery C. L.. (2023). Heart Disease and Stroke Statistics-−2023 Update: A Report From the American Heart Association. Circulation. 147, 112. 10.1161/CIR.0000000000001123 36695182 PMC12135016

[B65] Yacovino D. A. Akly M. P. Luis L. Zee D. S. (2018). The floccular syndrome: dynamic changes in eye movements and vestibulo-ocular reflex in isolated infarction of the cerebellar flocculus. Cerebellum. 17, 122–131. 10.1007/s12311-017-0878-1 28844105

